# The implied exploration-exploitation trade-off in human motor learning

**DOI:** 10.1186/1471-2202-12-S1-P98

**Published:** 2011-07-18

**Authors:** Holly N Phillips, Nikhil A Howai, Guy-Bart V Stan, Aldo A Faisal

**Affiliations:** 1 Department of Bioengineering, Imperial College London, London, SW7 2AZ, UK; 2 Centre for Synthetic Biology & Innovation, Imperial College London, London, SW7 2AZ, UK; 3 Department of Computing, Imperial College London, London, SW7 2AZ, UK

## 

The exploration-exploitation trade-off is at the heart of any sequential decision-making process in uncertain environments, such as foraging for food [1] or learning to cycle. This trade-off characterises the balance between reaping the benefits of a known solution (which may or may not be optimal) and continuing to search in hope for better solutions. It is difficult to directly infer or measure this trade-off from behavioural data in humans (or animals) on a trial-by-trial basis. Here, we use a set of reinforcement learning (RL) algorithms as ideal actor models to infer the internal representation of this trade-off from behavioural data. RL algorithms are well known to predict essential features of human and animal learning behaviour, and moreover these algorithms were shown to have direct electrophysiological and molecular correlates (such as reward and error learning signals) (see [2]).

To demonstrate our approach we conducted motor learning experiments based on a preliminary set of N=5 right-handed subjects (20-30 years of age). Our task consisted of computer-based psychophysics experiments (4 blocks of 50 trials) using a small grid world (5 states in 2x2 arrangements of states with a surrounding terminal state) in which subjects were to reach from their starting position to a goal state (Reward “$10”) while avoiding the terminal state (Reward “-$10”). Subjects were allowed to act in this grid world by moving in 4 cardinal directions under stochastic dynamics (actions had in each block probabilities of 0.7,0.8,0.9 or 1 a probability of moving in the chosen direction). However, to exclude human context knowledge that RL algorithms do not possess, we represented states by colours (so as to mask the spatial structure of the world). Subjects moved either in the form of unlabelled button presses or abstract gestures on a Wii Remote (NINTENDO, Kyoto, Japan). We found that both humans and our RL algorithms (TD, Q-Learning) required nearly the same amount of episodes to reach comparable performance.

The exploration-exploitation trade-off is formalised as a fundamental parameter of our two model-free RL algorithms, the so-called epsilon-greediness (which is the probability with which the learner will choose a sub-optimal action to explore other solutions). We inferred the implied human exploration-exploitation trade-off parameter by directly imposing the human state-action pairs on the algorithms. This allowed us to infer the internal representation of the subject’s optimal policy as well as the epsilon-greediness parameter (under the assumption that humans are learning using the corresponding algorithms based on the information available up to that episode). For example, we find for the task where the stochastic dynamics probability was 0.8, which the epsilon-greediness of human subjects increased rapidly in an initial exploration-intensive phase to values of 0.55 over the first 10 episodes. Once a near optimal solution was found, their epsilon-greediness decreased rapidly and stabilised back around 0.3.

These results show how we can gain insight into important parameters of reward-based learning problems from behavioural data. This approach may allow neurophysiologists to find neuronal correlates of exploration-exploitation trade-offs in the nervous system in sequential decision making tasks.
